# Treatment of Periarticular Fractures of the Knee Using the Less Invasive Stabilization System: A Retrospective Clinical Trial

**DOI:** 10.7759/cureus.7773

**Published:** 2020-04-22

**Authors:** Cenk Ermutlu, Ferdi Göksel, Gökay Eken

**Affiliations:** 1 Orthopaedics, Bursa Uludag University School of Medicine, Bursa, TUR; 2 Orthopaedics and Traumatology, Özel Karadeniz Ereğli Anadolu Hospital, Zonguldak, TUR; 3 Orthopaedics and Traumatology, Bursa Acıbadem Hospital, Bursa, TUR

**Keywords:** femur fracture, tibia fracture, liss, hospital stay

## Abstract

Introduction

Periarticular fractures of the knee in adults are generally treated with internal fixation. The Less Invasive Stabilization System (LISS) plate, developed by Arbeitsgemeinschaft Osteosynthesefragen (AO)/Association for the Study of Internal Fixation (ASIF) in the late 1990s, allows reduction and biological fixation through smaller incisions without violating periosteal blood supply. It offers several advantages for the treatment of complex periarticular fractures of the knee. In this study, we have aimed to report the results of a single series of these fractures.

Materials and methods

Forty-eight patients with AO type 33 and AO type 41 periarticular knee fractures who were operated between 2009 and 2014 at a single institution were included in this retrospective study. Patient demographics, fracture epidemiology, intraarticular extension, concomitant injuries, American Society of Anesthesiologists (ASA) score, time to union, the average time from admission to surgery, and the mean time from operation to patient discharge were noted. The effect of patient and fracture-related factors on length of hospital stay were evaluated.

Results

The mean follow-up time was 23.7 (12-48) months. The average time from admission till surgery and from surgery till discharge was 10.2 (1-39) and 9.7 (2-35) days, respectively. The average time for union was 6.8 months. Femur fractures healed in mean 6.6 months whereas tibia fractures took 7.1 months to heal. Time from admission to surgery and postoperative hospital stay was longer in patients with higher ASA scores (p<0.01) and open fractures (p<0.001). Patients’ body mass index (BMI) and intraarticular extension of the fracture did not cause an increase in either preoperative or postoperative hospital stay (p>0.05). The presence of concomitant major injuries caused a delay in operation (p<0.05), whereas postoperative hospital stay was not different (p>0.05).

Conclusion

LISS plating provides good stability through a small incision, permits biological fracture healing, may be used in multifragmentary fractures and has low complication rates. It is a good alternative for the treatment of periarticular fractures of the knee.

## Introduction

Periarticular fracture of the knee is defined as a fracture occurring anywhere between the distal femur and the proximal tibia. Its incidence and prevalence vary among different populations and geographic regions [1]. The main aim of treatment is to restore mechanical alignment and to achieve anatomical reduction and stable fixation of the articular surface to promote early motion of the knee to prevent joint stiffness [2-3]. Despite the diversity of modern implants and stabilization techniques, non-union, failure of fixation, malalignment, infection, and knee stiffness remain as potential complications [3-5].

The Less Invasive Stabilization System (LISS) plate, developed by Arbeitsgemeinschaft Osteosynthesefragen (AO)/Association for the Study of Internal Fixation (ASIF) in the late-1990s, allows reduction and biological fixation through smaller incisions without violating periosteal blood supply. It was initially developed for supracondylar femur fractures but its use has been extended to tibia plateau fractures, some tibia shaft fractures, and subtrochanteric fractures [2,6-7]. Even though it looks like a conventional plate, biomechanical, it acts more like an external fixator, hence commonly referred to as an “internal-external fixator” [8-9].

There are a few published reports about the role of LISS in the treatment of periarticular fractures of the knee. In this study, we have aimed to report the results of a single series of these fractures.

## Materials and methods

**Figure 1:**
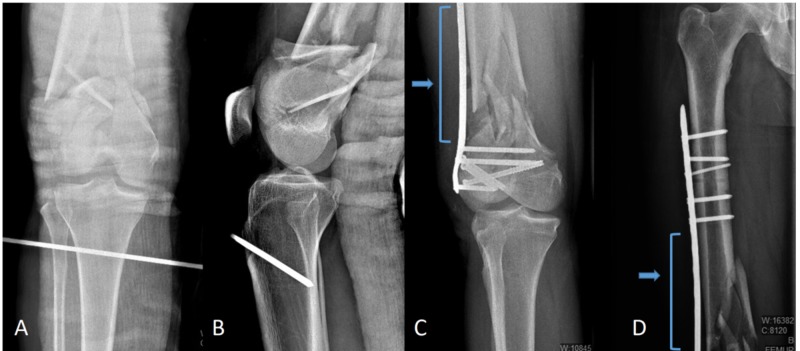
Fifty-year-old male patient with AO type 33-C3 fracture following a motor vehicle accident Arrows point to the section that would otherwise require extensive soft tissue dissection and possible periosteal stripping if biological fixation was not utilized. AO: Arbeitsgemeinschaft Osteosynthesefragen

**Figure 2:**
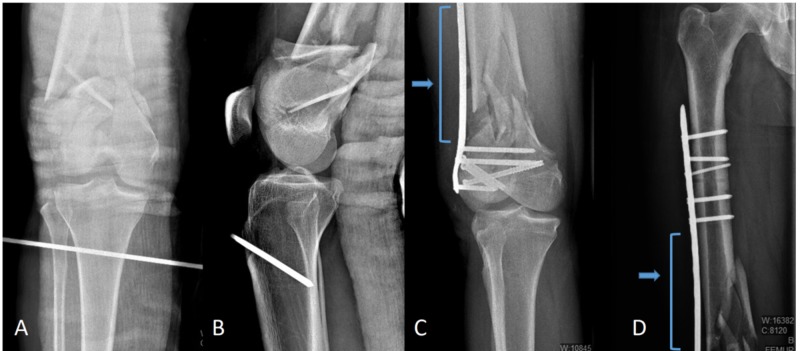
Fifty-two-year-old male patient with AO type 41-C1 fracture following a motorcycle accident Arrows point to the section that would otherwise require extensive soft tissue dissection and possible periosteal stripping if biological fixation was not utilized. AO: Arbeitsgemeinschaft Osteosynthesefragen

Following approval from the institutional review board (decision number 2020-1/51), records of patients with a periarticular knee fracture who were operated between 2009 and 2014 in our institution were retrospectively evaluated. Inclusion criteria were to be over 18 years of age and to have been treated with LISS plates. Patients with pathological or periprosthetic fractures and those with less than one-year follow-up were excluded from the study. All fractures were grouped according to the AO/OTA classification; AO type 33 (34 fractures) for the femoral side and AO type 41 (14 fractures) for tibia fractures (Figures 1A-1D and Figures 2A-2D). Twenty-nine fractures were closed whereas 19 were open. There were four Gustilo-Anderson type I, four type II, six type IIIB, and five type IIIC open fractures.

Patient demographics, fracture epidemiology, intraarticular extension, concomitant injuries, ASA score, BMI, time to union, the average time from admission to surgery, and mean time from operation to patient discharge were noted. The effect of patient and fracture-related factors on length of hospital stay were evaluated.

Closed fractures were provisionally stabilized by means of splinting in the emergency room. Open fractures were treated with splinting (six patients) or a spanning external fixator (13 patients) in the operating room following wound irrigation and debridement if necessary. Definitive treatment was performed once the patients’ overall condition was suitable for surgery.

The patients were operated on a radiolucent table in a supine position. After the fracture area was prepared, the lateral peripatellar approach was used for distal femur fractures and an anterolateral approach for proximal tibia fractures. Following adequate reduction, fractures were fixed using LISS plates with the minimally invasive technique. Thirty-two 13-hole, five 11-hole, nine nine-hole, and two seven-hole implants were used.

Active range of motion exercises were begun on the first day after surgery. Patients were called for follow-up at two, six, and 12 weeks. Partial weight-bearing was allowed once bridging callus was evident on any cortices on plain radiographs. Full weight-bearing was allowed at postoperative seven to 11 weeks. The mean follow-up time was 23.7 (12-48) months. Patients who had a bridging callus on three cortices and who could bear full weight without discomfort were considered to have complete union.

Statistical analysis

The normality of variables was examined using the Shapiro-Wilk test. The t-test or one-way analysis of variance (ANOVA) was used for between-group comparisons of variables with a normal distribution. The Tukey multiple comparison test was used for statistical significance. The level of significance was determined as α=0.05. Statistical analysis was performed using SPSS 23.0 (IBM SPSS Statistics for Windows, Version 23.0. IBM Corp., Armonk, NY) software.

## Results

Twenty-seven patients (56.2%) were male and 21 patients (43.2%) were female. The mean age was 48.1 years (range 18 to 75 years). The mean follow-up was 24 months (range 12-48). The cause of fracture was a simple fall in seven (14.6%) cases, motor vehicle accident in 22 (45.8%) cases, fall from a height in eight (16.7%) cases, fall of heavy objects in five (10.4%) cases, gunshot injury in five (10.4%) cases, and blunt trauma in one (2.1%) case. Three patients had contralateral AO 41-A type periarticular knee fractures, which were treated with other implants.

Of the 48 patients, 22 had additional injuries such as concomitant fractures, vascular injuries, or visceral injuries. Twenty-two (45.8%) patients were ASA I, 25 patients (52.1%) were ASA II, and one patient (2.1%) was ASA III. The mean operation length was 137 minutes. The average time from admission till surgery and from surgery till discharge was 10.2 (1-39) and 9.7 (2-35) days, respectively. The average time for union was 6.8 months. Femur fractures healed in mean 6.6 months whereas tibia fractures took mean 7.1 months to heal.

The average time from admission to surgery and the postoperative hospital stay were longer in patients with higher ASA scores (p<0.01) and open fractures (p<0.001). The patients’ BMI and intraarticular extension of the fracture did not cause an increase in either preoperative or postoperative hospital stay (p>0.05). The presence of concomitant major injuries caused a delay in operation (p<0.05), whereas postoperative hospital stay was not different.

Two patients with a distal femur fracture had malreduction on the sagittal plane while one patient with proximal tibia fracture had 10° of varus. All three patients were reluctant to undergo revision surgery because of frustration caused by the long recovery period they lived through after initial trauma.

Forty-one fractures healed without any complications. Surgical site infection occurred in two patients. One patient was a 59-year- old woman with a Gustilo-Anderson type IIIB (AO/OTA type 33C3) fracture and the other one was a 40-year-old man with a closed AO type 41C2 fracture. They were treated with surgical debridement combined with a course of intravenous antibiotics. No implant removal was necessary.

One patient (a 40-year-old man with a closed type 41C2) developed pulmonary embolism 11 days after surgery. He was transferred to the intensive care unit. The patient fully recovered and was admitted to the orthopedics ward after five days.

Implant (LISS)-specific complications occurred in one patient. A 59-years old woman with a Gustilo-Anderson type IIIB (AO/OTA type 33C3) fracture had a broken plate one month after the surgery. The broken plate was revised with the same size implant.

## Discussion

Periarticular fractures of the knee are not uncommon, and they can occur in any age group. Their age-specific prevalence differs among males and females. Fractures in males generally result from high-energy trauma at a younger age, whereas low-energy fractures tend to occur in older females with osteoporosis. Cases that are results of high-energy trauma are usually complicated with concomitant injuries that lengthen the hospital stay [1,10-11]. In our study, the rate of concomitant injuries was 45.8%, which is higher than other reports in the literature. This is largely due to the fact that the institution where this study was conducted is a regional university hospital where complex trauma cases are referred to.

Several methods have been described for the treatment of periarticular fractures of the knee. Each one has its advantages and disadvantages. LISS plates offer several advantages for the treatment of complex periarticular fractures of the knee. There are several studies reporting the outcome of LISS plates [2,5-9]. Following indirect closed reduction, the plate can be advanced into the submuscular space through a small lateral incision [12]. The anatomically contoured form of the plate allows easy placement alongside the lateral distal femur or proximal tibia [9,13]. Among other options for supracondylar femur fractures are intramedullary nailing (IMN), condylar buttress plates or dynamic condylar screws. Although biomechanical studies have proved that IMN and plate constructs have approximate axial strength, IMNs may fail with axial loading. Furthermore, IMNs are not suitable for intraarticular comminuted fractures of this area. The need for arthrotomy is also another potential drawback of IMN use in this area. As for the condylar buttress plates and dynamic condylar screws, extensive dissection is necessary to place and fix the plates, thus increasing blood loss, periosteal stripping, and risk of infection. Another advantage of LISS plating is the faster fracture healing time due to avoiding periosteal stripping and hematoma drainage [4-5]. Also, LISS plates resist higher loads and provide more stable fixation when compared to conventional plating, especially in osteoporotic bone [5,14-15].

LISS plates may also be used for cases with nonunion, periprosthetic fractures, and severe osteoporosis. Although these plates no longer offer the advantage of a minimally invasive application when treating nonunion, it is still possible to achieve high rates of union [8-9,13]. They are recommended over IMNs and other plates for the treatment of periprosthetic fractures due to minimal invasive application, mechanical advantages, and ease of application, with satisfactory results [16-17].

LISS plates may be used for fractures of several other anatomical regions. Li et al. have treated 26 patients with unstable subtrochanteric femur fractures using contralateral reverse LISS and achieved primary union without any complications [18]. Similarly, Lewis et al. have reported the successful use of a reverse LISS plate on a 36-year-old female patient with a Gustilo and Anderson Type III B open femur shaft fracture with an intertrochanteric component [19]. Lui et al. have published the results of a contralateral LISS plate application for proximal tibia fractures in five patients [2].

Open fractures of the knee region are not uncommon. In our study, even though the percentage of patients with open fractures and multiple trauma was high, LISS plate application yielded union times similar to other studies in the literature and no nonunion occurred [1-3,6-9]. An open fracture is also more prone to soft tissue infection and resultant osteomyelitis. When performed after good wound care, antibiotic administration, and temporary external fixation, the definitive treatment of open fractures with LISS plates through small incisions results in low osteomyelitis incidence [2,6]. Similarly in our study, there were just two cases of superficial surgical site infections and no osteomyelitis occurred.

Despite its advantages, implant failure is still a risk as it is for other implants. It often occurs in older patients and patients with osteoporosis or periprosthetic fractures who are prone to other complications as well [2-3,12,20]. In their study on 189 periarticular fractures of the knee, Liu et al. have reported three implant failures [2]. Muller et al. have reported four such cases during the treatment of periprosthetic fractures [20]. In Smith et al.'s review of periarticular fractures of the knee, 11 LISS plates out of 694 had failed. In our series, one patient with an AO/OTA type 33C3 fracture had a broken implant, which was revised with a LISS plate again [3].

Some studies have reported the pull-out of proximal screws. This complication is thought to occur because of unicortical screwing, short plate use, and early weight-bearing. Avoiding short plates, using bicortical screwing, and delaying weight-bearing till bridging callus is evident may prevent this complication [4,8].

This study has the limitations of a retrospective study design. The retrospective design and possible effects of confounding variables, such as osteoporosis, smoking, hormonal imbalance, and corticosteroid or nonsteroidal anti-inflammatory drug (NSAID) use, which were not evaluated, are among the limitations of our study.

## Conclusions

LISS plating provides good stability through a small incision, permits biological fracture healing, may be used in multifragmentary fractures, and has low complication rates, making it a good alternative for the treatment of periarticular fractures of the knee. It has low rates of surgical site infections, osteomyelitis, and nonunion even in the treatment of open fractures. Even though the presence of additional injuries causes a delay in surgery, the postoperative recovery is similar to cases with isolated unilateral fractures. The BMI and complexity of the fracture had no effect on preoperative or postoperative hospital stay.
